# Co-delivery of docetaxel and endostatin by a biodegradable nanoparticle for the synergistic treatment of cervical cancer

**DOI:** 10.1186/1556-276X-7-666

**Published:** 2012-12-06

**Authors:** Bo Qiu, Minghui Ji, Xiaosong Song, Yongqiang Zhu, Zhongyuan Wang, Xudong Zhang, Shu Wu, Hongbo Chen, Lin Mei, Yi Zheng

**Affiliations:** 1 Department of Orthopedics, Renmin Hospital of Wuhan University, Wuhan, 430060, People’s Republic of China; 2 Southern Medical University, Guangzhou, 510515, People’s Republic of China; 3 The Shenzhen Key Lab of Gene and Antibody Therapy, Graduate School at Shenzhen, Tsinghua University, Shenzhen, 518055, People’s Republic of China; 4 School of Life Sciences, Tsinghua University, Beijing, 100084, People’s Republic of China; 5 L401, Tsinghua Campus, Xili University Town, Shenzhen, Guangdong Province, 518055, China

**Keywords:** TPGS-*b*-(PCL-*ran*-PGA), nanoparticles, cervical cancer, endostatin, docetaxel

## Abstract

Cervical cancer remains a major problem in women's health worldwide. In this research, a novel biodegradable d-α-tocopheryl polyethylene glycol 1000 succinate-*b*-poly(ε-caprolactone-*ran-*glycolide) (TPGS-*b*-(PCL-*ran*-PGA)) nanoparticle (NP) was developed as a co-delivery system of docetaxel and endostatin for the synergistic treatment of cervical cancer. Docetaxel-loaded TPGS-*b*-(PCL-*ran*-PGA) NPs were prepared and further modified by polyethyleneimine for coating plasmid pShuttle2-endostatin. All NPs were characterized in size, surface charge, morphology, and *in vitro* release of docetaxel and pDNA. The uptake of coumarin 6-loaded TPGS-*b*-(PCL-*ran*-PGA)/PEI-pDsRED by HeLa cells was observed via fluorescent microscopy and confocal laser scanning microscopy. Endostatin expression in HeLa cells transfected by TPGS-*b*-(PCL-*ran*-PGA)/PEI-pShuttle2-endostatin NPs was detected using Western blot analysis, and the cell viability of different NP-treated HeLa cells was determined by MTT assay. The HeLa cells from the tumor model, nude mice, were treated with various NPs including docetaxel-loaded-TPGS-*b*-(PCL-*ran*-PGA)/PEI-endostatin NPs, and their survival time, tumor volume and body weight were monitored during regimen process. The tumor tissue histopathology was analyzed using hematoxylin and eosin staining, and microvessel density in tumor tissue was evaluated immunohistochemically. The results showed that the TPGS-*b*-(PCL-*ran*-PGA)/PEI NPs can efficiently and simultaneously deliver both coumarin-6 and plasmids into HeLa cells, and the expression of endostatin was verified via Western blot analysis. Compared with control groups, the TPGS-*b*-(PCL-*ran*-PGA)/PEI-pShuttle2-endostatin NPs significantly decreased the cell viability of HeLa cells (*p* < 0.01), inhibited the growth of tumors, and even eradicated the tumors. The underlying mechanism is attributed to synergistic anti-tumor effects by the combined use of docetaxel, endostatin, and TPGS released from NPs. The TPGS-*b*-(PCL-*ran*-PGA) NPs could function as multifunctional carrier for chemotherapeutic drugs and genetic material delivery, and offer considerable potential as an ideal candidate for *in vivo* cancer therapy.

## Background

Cervical cancer caused by high-risk human papillomavirus (HPV) persistent infection is the second most common cancer in women worldwide [1,2]. Fortunately, two HPV vaccines (Gardasil (Merck Sharp & Dohme Corp., NJ, USA) and Cervarix (GlaxoSmithKline, TW, UK)) have been approved for use in many countries that would effectively reduce the incidence of cervical cancer genesis. However, these two vaccines have not shown any therapeutic effect against current cervical cancer, HPV infections or associated lesions [3]. Thus, there are urgent needs to develop new specific drugs or novel therapeutic methods for cervical cancer treatment.

It has been well established that the progression of solid tumors, including cervical cancer, is critically dependent on neoangiogenesis for nutrition and oxygen supply. Therefore, blockade of neoangiogenesis has been regarded as a promising strategy for tumor therapy [4-8]. Endostatin, a 20 kDa C-terminal proteolytic fragment of collagen XVIII, has received the greatest attention for its broad-spectrum and low-toxic anti-angiogenesis ability [6,9,10]. These advantages accelerate the investigation process of endostatin into the clinical trial [11,12]. However, as a protein, endostatin has many challenges in its clinical application, such as short half-life and instability. Recently, gene therapy, the use of DNA as a pharmaceutical agent, has been extensively studied in a broad range of diseases including tumors, which can achieve a relative long-term stable expression of therapeutic proteins [13-16]. A major limitation of gene therapy is the efficient delivery of therapeutic DNA to the target cells and tissues. The main vectors for DNA delivery are viral vectors and non-viral vectors having great advantages in safety, convenient large-scale production, physiological stability, and no immunogenicity.

Currently, a variety of particulate drug delivery system based on synthetic and natural materials has been investigated as DNA carriers, including liposomes [17], dendrimers [18], polycationic polymers [19,20], and polymeric nanoparticles (NPs) [21]. Among them, PEI-based NPs are considered as one of the most effective carrier since it can complex with pDNA or siRNA and exhibit a unique ‘proton sponge effect’ for endosomal release of the nano-complexes into cytosol [22]. On the other hand, many chemotherapeutic drugs such as paclitaxel, doxorubicin and mitomycin are also limited because of low water solubility, acute toxicity to normal tissues due to lack of targeting, and multi-drug resistance. Several researches showed that the combination of endostatin and chemotherapeutic drugs such as docetaxel can result in better inhibitory effects for tumor growth than single therapy [23]. However, the clinical applications of these combined drugs are needed to develop new efficient drug delivery system.

A novel biodegradable copolymer d-α-tocopheryl polyethylene glycol 1000 succinate-*b*-poly(ε-caprolactone-*ran-*glycolide) (TPGS-*b*-(PCL-*ran*-PGA)) has been successfully synthesized in our laboratory [24]. Our previous research has confirmed that docetaxel-loaded TPGS-*b*-(PCL-*ran*-PGA) NPs can be efficiently uptaken by MCF-7 cells and effectively inhibit the growth of tumor over a longer period of time than commercial Taxotere® (Sanofi-Aventis US LLC, NJ, USA) at the same dose. To overcome the limitations of single therapy, we modified the TPGS-*b*-(PCL-*ran*-PGA) NPs with a polyplexed PEI and evaluated its ability to simultaneously deliver DNA and a chemotherapeutic drug in cells. Most importantly, we examined its therapeutic effect on xenograft models bearing cervical cancer cells.

## Methods

### Polymers and reagents

TPGS-*b*-(PCL-*ran*-PGA) copolymer (Mw approximately 24,000) was synthesized in our laboratory (Tsinghua University, China). Poly (vinyl alcohol) (PVA; (80% hydrolyzed), dichloromethane, branched polyethylenimine (MW approximately 25,000, BPEI25k) and 3-(4,5-dimethylthiazol-2-yl)-2,5-diphenyltetrazolium bromide (MTT) kit were purchased from Sigma-Aldrich Corporation (MO, USA). 4',6-diamidino-2-phenylindole (DAPI) was purchased from VECTOR (Burlingame, USA). Dulbecco's Modified Eagles' Medium (DMEM), penicillin-streptomycin, fetal bovine serum (FBS), and Dulbecco's phosphate buffered saline (DPBS) were purchased from Invitrogen-Gibco (Carlsbad, CA). Plasmid vectors pShuttle2, pIRES-EGFP, and pDsRED-E1 were acquired from Invitrogen Corporation (Clontech, USA).

### Preparation of expression vectors

Human endostatin gene was amplified by PCR using the following primers: hEndostatin-F: 5^′^-GCTCTAGA(XbaІ)gccaccatgggaattcatgcacagccaccgcgacttcc-3^′^ and hEndostatin-R: 5^′^-GGGGTACC (KpnІ)ttacttggaggcagtcatg-3^′^. PCR products were digested with Xba I and KpnІ and inserted into pShuttle2 vector (Clontech). The recombinant plasmid pShuttle2-endostatin was verified by DNA sequencing. The expression of endostatin in HeLa cells transfected with PEI or TPGS-*b*-(PCL-*ran*-PGA)-based NPs was analyzed by Western blot analysis.

### Preparation of docetaxel-loaded TPGS-*b*-(PCL-*ran*-PGA) NPs and determination of drug contents

Docetaxel-loaded and blank TPGS-*b*-(PCL-*ran*-PGA) NPs were prepared using a modified water-in-oil-in-water solvent evaporation as described previously [25]. Briefly, TPGS-*b*-(PCL-*ran*-PGA) copolymer (50 mg) and a certain amount of docetaxel (0 to 25mg) were dissolved in 2 ml methylene chloride, followed by 30-s sonication to emulsify the mixture (UW 70/HD 70; tip, MS 72/D; Bandelin Electronic GmbH & Co., Berlin, Germany). After the addition of 3 ml of 7% (*w*/*v*) aqueous solution of PVA, the emulsion was sonicated again for 20 s. The resulting double emulsion was then poured into 50 ml 1% (*w*/*v*) aqueous PVA solution containing 2% isopropanol and then maintained under mechanical stirring for 1 h at 600 rpm. The residual methylene chloride was then evaporated by vacuum. Next, the 2 ml aliquots of the nanosphere suspension were washed twice with 20 mM 4-(2-hydroxyethyl)-1-piperazineethanesulfonic acid (HEPES)/NaOH (pH 7.0), and the NPs were harvested by centrifugation at 7,000 rpm for 10 min. In addition, the fluorescent coumarin-6 loaded TPGS-*b*-(PCL-*ran*-PGA) NPs were prepared in the same way except that 0.1% (*w*/*v*) coumarin-6 was entrapped instead of docetaxel.

The docetaxel-loading content, defined as the mass percentage of docetaxel entrapped in nanoparticles, was quantified using UV–vis analysis (UV-2000 UV–vis spectrophotometer, Unico Inc., WI, USA). First, docetaxel-loaded nanoparticle solutions were lyophilized to yield the solid nanoparticle samples. Then, the dried nanoparticle samples were weighed and redissolved in a mixture of chloroform and DMSO (1:1, *v*/*v*). The absorbance of docetaxel at 482.5 nm was measured, and the drug content in the solution was calculated based on a previously established calibration curve.

### Preparation of PEI-modified TPGS-*b*-(PCL-*ran*-PGA) NPs/DNA complexes

The NPs were prepared as described previously [25]. Briefly, the particles were formulated with a ratio of the polymer nitrogen to the DNA/phosphate (N/P) equal to TPGS-*b*-(PCL-*ran*-PGA) NP solution (0.2 ml) and were mixed with 2 mg of PEI in sterile HEPES-buffered saline. The PEI-modified TPGS-*b*-(PCL-*ran*-PGA) NP solution was then added to the plasmid DNA solution at different N/P ratios and vortexed gently. The PEI-modified TPGS-*b*-(PCL-*ran*-PGA) NP/DNA complexes were incubated in sterile PBS for 20 min at room temperature.

### Characterization of nanoparticles

The mean particle size and size distribution were measured using dynamic light scattering (DLS) (Zetasizer Nano ZS90, Malvern Instruments Ltd., Malvern, UK). In brief, the NPs were suspended in deionized water at a concentration of 0.1 mg/ml. The mean hydrodynamic diameter was determined via cumulative analysis. The DLS determinations were predicated based on the electrophoretic mobility of the NPs in an aqueous medium. The electrophoretic mobility was evaluated using folded capillary cells in an automatic mode. Zeta potential of the NPs was detected using laser Doppler anemometry (LDA; Zetasizer Nano ZS90, Malvern Instruments Ltd.). Samples were prepared in PBS and diluted 1:3 with deionized water to ensure that the measurements were performed under conditions of low ionic strength where the surface charge of the particles can be measured accurately. The final concentration of the polymer was 0.01 mg/ml. All data represent five measurements from one sample.

### Gel retardation assay

The binding of pDNA with free TPGS-*b*-(PCL-*ran*-PGA)/PEI NPs was determined by 0.8% agarose gel electrophoresis. A series of different weight ratios (*w*/*w*) of pDNA to NPs was loaded on the agarose gel (10 μl of the sample containing different amounts of pDNA). The pDNA bands were then visualized under a UV transilluminator at a wavelength of 365 nm.

### Evaluation of loading efficiency of pDNA to the NPs

The loading efficiency of pDNA to the NPs was evaluated by measuring unbound pDNA in the NPs solution. The supernatant in the loaded docetaxel or free TPGS-*b*-(PCL-*ran*-PGA)/PEI NPs solution was recovered by removing the NPs by centrifugation. Free pDNA concentration in the supernatant was determined by measuring absorbance at 260 nm using a UV-2550 spectrophotometer (Shimadzu Corp., Kyoto, Japan). Loading efficiency of pDNA in NPs was determined by subtracting the amount of pDNA recovered in the washing solutions from initial amount of pDNA added. Encapsulation efficiency was defined as the percentage of pDNA encapsulated in NPs with respect to the initially added amount of pDNA.

### *In vitro* drug release

NPs with 15 mg docetaxel-loaded TPGS-*b*-(PCL-*ran*-PGA) were dispersed in 5 ml release medium (phosphate buffer solution of pH 7.4 containing 0.1% *w*/*v* Tween 80) to form a suspension. Tween 80 was used to increase the solubility of docetaxel in the buffer solution and avoid the adherence of docetaxel to the tube wall. The suspension was transferred into a dialysis membrane bag (Spectra/Por 6, MWCO = 1,000, Spectrum Laboratories, Inc., TX, USA). Then, the closed bag was put into a centrifuge tube and immersed in 15-ml release medium. The tube was put in an orbital water bath at 37°C under a 120 rpm horizontal shaking. Ten milliliters of solution was periodically removed for analysis and was replaced with fresh release medium. The collected samples were mixed in a mixture of chloroform and DMSO (1:1, *v*/*v*). The analysis procedure was the same as described above.

To investigate the *in vitro* release of pDNA, 5 mg of TPGS-*b*-(PCL-*ran*-PGA)/PEI-25K (*n* = 6) and coumarin-6-loaded TPGS-*b*-(PCL-*ran*-PGA)/PEI (*n* = 6) were incubated in 1 ml of DPBS buffer (pH 7.4) in a micro-centrifuge tube in shaking incubator at 37°C. After incubating for 24 h, half of the samples (*n* = 3) were transferred into a 25 mM sodium acetate buffer (pH 5.0) to simulate acidication of the endolysosome of the cell. Samples were taken periodically in microcentrifuge tubes and were centrifuged at 14,000 rpm for 10 min to obtain pellet NPs. The supernatants were removed and replaced with fresh buffer, and NPs were resuspended by vigorous pipetting. The supernatants were stored at −70°C until analysis by UV measurement. Unmodified TPGS-*b*-(PCL-*ran*-PGA) NPs were also analyzed as a background control.

### Cell culture

HeLa and 293T cells (ATCC, VA, USA) were cultured in DMEM, pH 7.4, supplemented with 25 mM NaHCO_3_, 10 μg/ml streptomycin sulfate, 100 μg/ml penicillin G, and 10%(*v*/*v*) FBS. Cells were maintained at 37°C in a 5% CO_2_, 95% air incubator.

### Cell viability

The cytotoxicity of the NPs was determined using MTT assay. The culture medium was removed and replaced with 20 μl/well MTT (5 mg/ml) solution in each well, followed by 4-h incubation at 37°C in a fully humidified atmosphere with 5% CO_2_. MTT was taken up by active cells and transformed into insoluble purple formazan granules in the mitochondria. Subsequently, the medium was discarded, the precipitated formazan was dissolved in DMSO (150 μl/well), and optical density of the solution was evaluated using a microplate spectrophotometer at a wavelength of 570 nm. The analytic assays were performed every day, and at least 4 wells were randomly taken into examination each time. The determination of cell viability depends on these physical and biochemical properties of cells.

### Cellular uptake of PEI-modified TPGS-*b*-(PCL-*ran*-PGA) NPs

The cells were plated in a 6-well (3 × 10^5^ per well) plate, cultured at 37°C in 5% CO_2_, rinsed twice, and pre-incubated for 1 h with 2 ml of serum-free medium at 37°C. Coumarin-6-loaded TPGS-*b*-(PCL-*ran*-PGA)/PEI NPs with or without pDsRED or pIRES-EGFP were added in a particle concentration of 0.01 to 0.2 mg/ml and incubated for 1 to 4 h at 37°C. The cells were then washed three times with 1 ml of PBS (pH 7.4) to remove free PEI/plasmids-modified TPGS-*b*-(PCL-*ran*-PGA) NPs, resuspended in PBS, and then analyzed by fluorescent microscopy.

For confocal laser microscopy analysis, cells were pre-incubated for 1 h at 37°C in serum-free medium and then for 30 min to 4 h in the presence of PEI/plasmids pIRES-EGFP or/and pDsRED gene-modified TPGS-*b*-(PCL-*ran*-PGA) NPs with a final particle concentration of 0.05 mg/ml. The samples were mounted in fluorescent mounting medium (Dako, Glostrup, Denmark), and fluorescence was monitored. Images were acquired using a confocal laser microscope (Eclipse TE 2000, Nikon Corporation, Tokyo, Japan).

### Western blot

The cells were seeded into 6-well plates overnight, allowed to attach the bottom. Cells were cultured at 37°C in 5% CO_2_, rinsed twice, and pre-incubated for 1 h with 2 ml of serum-free medium at 37°C. Endostatin gene-modified TPGS-*b*-(PCL-*ran*-PGA) NPs were added in a particle concentration of 0.01 to 0.2 mg/ml and incubated for 1 to 4 h at 37°C. The cells were then washed three times with 1 ml PBS (pH 7.4) to remove any free PEI/plasmid-modified TPGS-*b*-(PCL-*ran*-PGA) NPs. After being cultured in fresh complete medium for 48 h, the cells were lysed with lysis buffer (Beyotime Institute of Biotechnology, Haimen, Jiangshu, China) containing PMSF (Sigma Chemical Co., St. Louis, MI, USA) for 30 min at 4°C. The lysate was then centrifuged for 20 min at 13,000 rpm and 4°C. The proteins were then separated through SDS-PAGE and transferred onto the PVDF membrane (Immobion-P Transfer Membrane, Millipore Corp., Billerica, MA, USA). Membranes were blocked in a tris-buffered saline with 0.1% Tween 20 solution containing 5% non-fat dry milk and incubated overnight with anti-human endostatin antibody as primary antibody at 4°C. Anti-β-actin antibody was used as loading control.

### Mice maintenance and subcutaneous tumor model

Female BALB/c-nu/nu mice were supplied by the Medical Experimental Animal Center of Guangdong Province (Guangdong, China). A subcutaneous tumor model of nude mice was constructed. Six-week-old female nude mice (18 ± 2 g) were subcutaneously inoculated with 1.5 to 2 × 10^6^ HeLa cells. When the tumor size reached about 100 mm^3^, the mice were randomly divided into six groups with six mice each: PBS, TPGS-*b*-(PCL-*ran*-PGA) (group ANP), TPGS-*b*-(PCL-*ran*-PGA)/PEI(group BNP), TPGS-*b*-(PCL-*ran*-PGA)/PEI-pEndostatin (group CNP), docetaxel-loaded TPGS-*b*-(PCL-*ran*-PGA) (group DNP), and docetaxel-loaded TPGS-*b*-(PCL-*ran*-PGA)/PEI-pEndostatin (group FNP), of which each dose of NPs contained 0.2 mg PCL-PGA-TPGS, 10 μg PEI, and 50 μg pDNA for treatment. The groups were treated once every 3 days with intratumoral injections of PBS or NPs. Tumor size was measured with a caliper, and the weight of each mouse was measured with a scale. Tumor volume was calculated as the equation ‘volume = length × width^2^ / 2’. The mean tumor volume and mouse weight were used to construct the curves of tumor growth versus mouse growth to evaluate therapeutic efficiency and toxicity.

At the end of the treatment, the mice in each group were killed and dissected. The heart, liver, lungs, spleen, kidneys, and other organs were checked for signs of toxicity. Tumor specimens were then prepared as paraffin-embedded sections for histopathological analysis. The study was carried out in strict accordance with the recommendations in the Guide for the Care and Use of Laboratory Animals of Institutional Animal Care and Use Committee of Graduate School at Shenzhen, Tsinghua University. The protocol was approved by the Animal Welfare and Ethics Committee of Graduate School at Shenzhen, Tsinghua University, China (no. 2011-YZ-BG).

### Measurement of microvessel density in tumor tissues

Microvessel density (MVD) in tumor tissue was evaluated immunohistochemically using a monoclonal anti-mouse CD31 antibody (rat anti-mouse CD31 monoclonal antibody, clone MEC 13.3; BD Biosciences, NJ, USA). Tumor samples were collected after the therapy regimen was finished. Immunohistochemical staining was performed as described previously [26]. MVD (%) was calculated from the ratio of the CD31-positive staining area to the total observation area in the viable region. Three to six fields per section were randomly analyzed, excluding necrotic areas. Positive staining areas were calculated using imaging analysis software (Win Roof; Mitani Corporation, Fukui, Japan).

### Statistics and data analysis

All cell experiments were repeated at least three times unless otherwise indicated. Images of Western blot results from representative experiments were presented. The figures were created using Adobe Photoshop CS graphics program. All data were analyzed by paired *t* test using SPSS 11.0 software. Differences were considered statistically significant at *P* < 0.05.

## Results and discussions

### The expression of pShuttle2-endostatin

**Figure 1:**
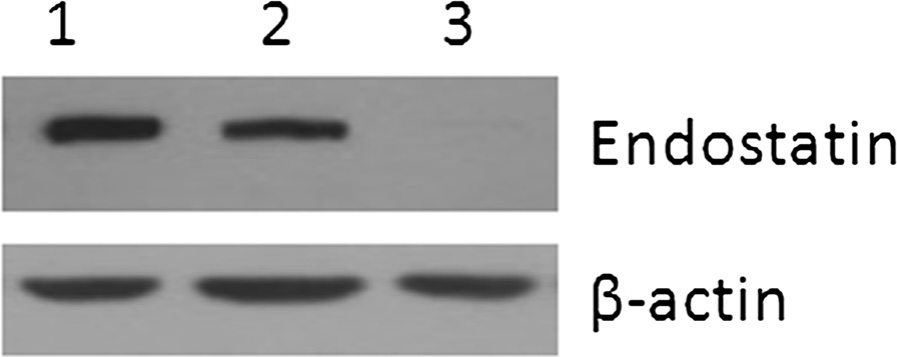
**Expression of endostatin in HeLa cells using Western blot analysis.** Lane 1: HeLa cells were transfected by pShuttle2-endostatin using PEI, lane 2: transfected by pShuttle2-endostatin using TPGS-*b*-(PCL-*ran*-PGA)/PEI, lane 3: transfected by pShuttle2 using TPGS-*b*-(PCL-*ran*-PGA)/PEI.

Protein expression of endostatin was analyzed by Western blot using cell lysate after transfection of HeLa cells using PEI (Figure 1). These results showed that pShuttle2-endostatin was successfully constructed and expressed in HeLa cells.

### Characterization of nanoparticles

**Figure 2:**
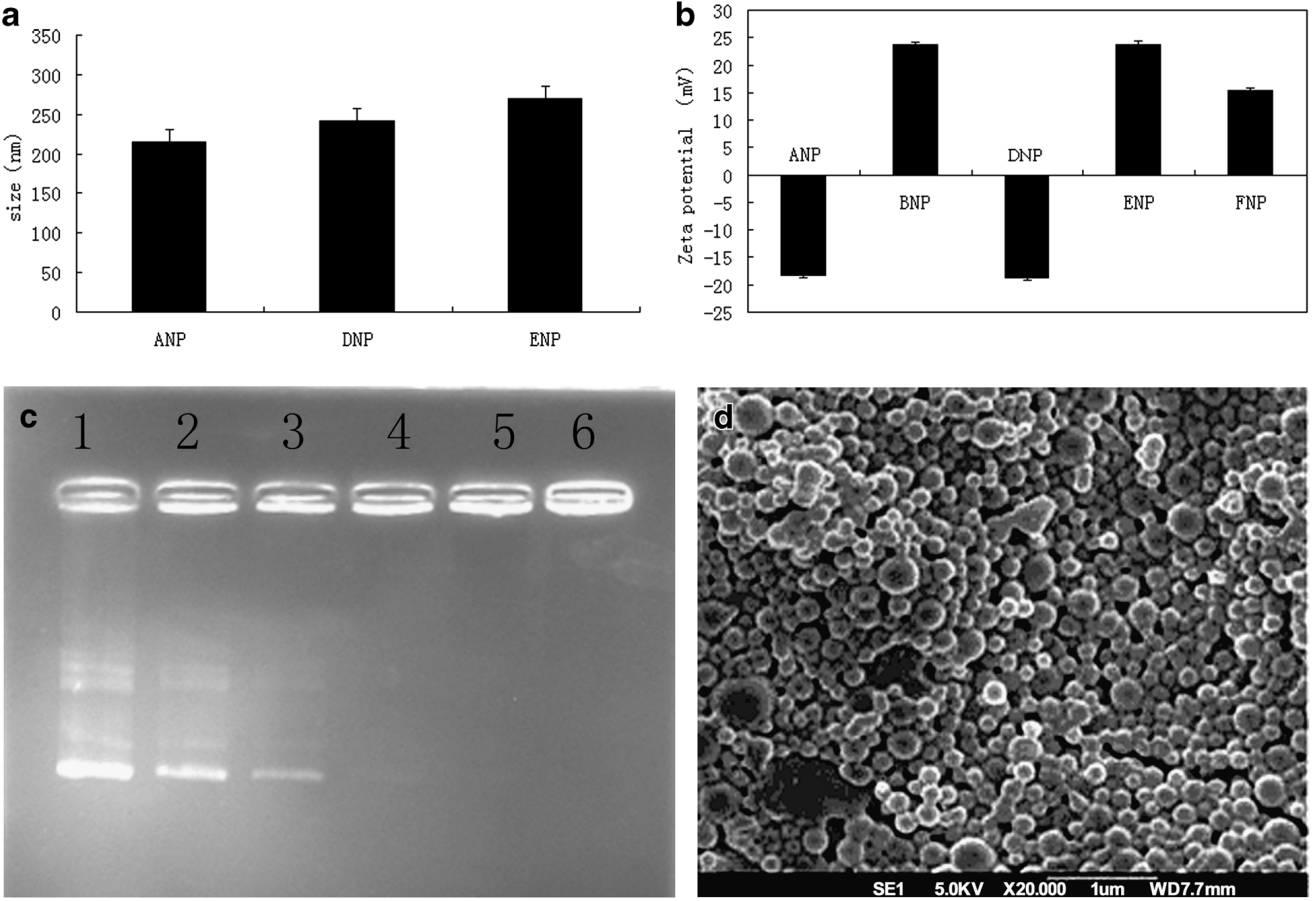
**The size of different NPs (mean ± SD) (A).** ANPs: TPGS-*b*-(PCL-*ran*-PGA) NPs; DNPs: docetaxel-loaded TPGS-*b*-(PCL-*ran*-PGA) NPs; ENPs: docetaxel-loaded TPGS-*b*-(PCL-*ran*-PGA)/PEI. The results showed that the size of NPs increased because of loading docetaxel and/or being modified by PEI. The zeta potential of the different NPs (**B**). The zeta potential of blank NPs was negative; the zeta potential of the NPs modified by PEI was positive and reduced after complexed with pDNA. The results showed that TPGS-*b*-(PCL-*ran*-PGA) blank NPs could be modified. Gel retardation assay of pDNA complexed with TPGS-*b*-(PCL-*ran*-PGA)/PEI (**C**). Each lane contained the same pDNA. Surface morphology of the PEI-modified TPGS-*b*-(PCL-*ran*-PGA) NPs was examined by FESEM (**D**).

Recently, a novel biodegradable copolymer, TPGS-*b*-(PCL-*ran*-PGA), has been successfully synthesized in our laboratory [24]. Our previous study showed that the NPs made of this novel copolymer can improve drug permeability, solubility, degradation, and avoid the disadvantages of single component [24]. More importantly, TPGS, the degradation products of TPGS-*b*-(PCL-*ran*-PGA), showed synergistic anticancer activity in the presence of anticancer agents [24]. In this research, to co-delivery chemotherapeutic drugs and genetic materials into cells, TPGS-*b*-(PCL-*ran*-PGA) NPs were modified with cationic polymer PEI (25 K). DLS assay showed that the hydrodynamic diameter of the polyplexed blank TPGS-*b*-(PCL-*ran*-PGA) NPs (ANPs) was approximately 215 nm, while the diameters of the docetaxel-loaded TPGS-*b*-(PCL-*ran*-PGA) and docetaxel-loaded TPGS-*b*-(PCL-*ran*-PGA) PEI-coated without pDNA NPs were approximately 242 and approximately 270 nm, respectively (Figure 2A). The FESEM images showed that the morphologies of TPGS-*b*-(PCL-*ran*-PGA)/PEI NPs were sphere-like in shape, and the size was about 250 nm (Figure 2D), consistent with the DLS data. The result suggested that PEI modification may increase the size of the NPs from TPGS-*b*-(PCL-*ran*-PGA) copolymer. In addition, the surface charge of the unmodified TPGS-*b*-(PCL-*ran*-PGA) NPs was approximately −18.25 mV detected by LDA assay, while the surface charge of the PEI-modified NPs was significantly increased in zeta potential, suggesting that the surface charge of the TPGS-*b*-(PCL-*ran*-PGA) NPs was altered from negative to positive due to the PEI coating. However, a reduction in surface charge of the PEI-modified NPs was observed after adding pDNA, suggesting PEI condenses negatively charged DNA into positively charged particles (Figure 2B). Agarose gel electrophoresis assay showed that the amount of NP-binding DNA increased as the amount of TPGS-*b*-(PCL-*ran*-PGA)/PEI NPs increased (Figure 2C). In a recent report, the DNA encapsulated in the polymeric (PLGA) NPs is in a condensed form, which could protect itself from denaturation and allow it to be efficiently taken up by MSCs [25]. Another study also showed that PLGA/PEI NPs can protect DNA against degradation and reduce the cytotoxicity caused by PEI [25]. Together, results showed that the TPGS-*b*-(PCL-*ran*-PGA) NPs modified by PEI could encapsulate docetaxel and DNA with a high loading efficiency.

### *In vitro* release

**Figure 3:**
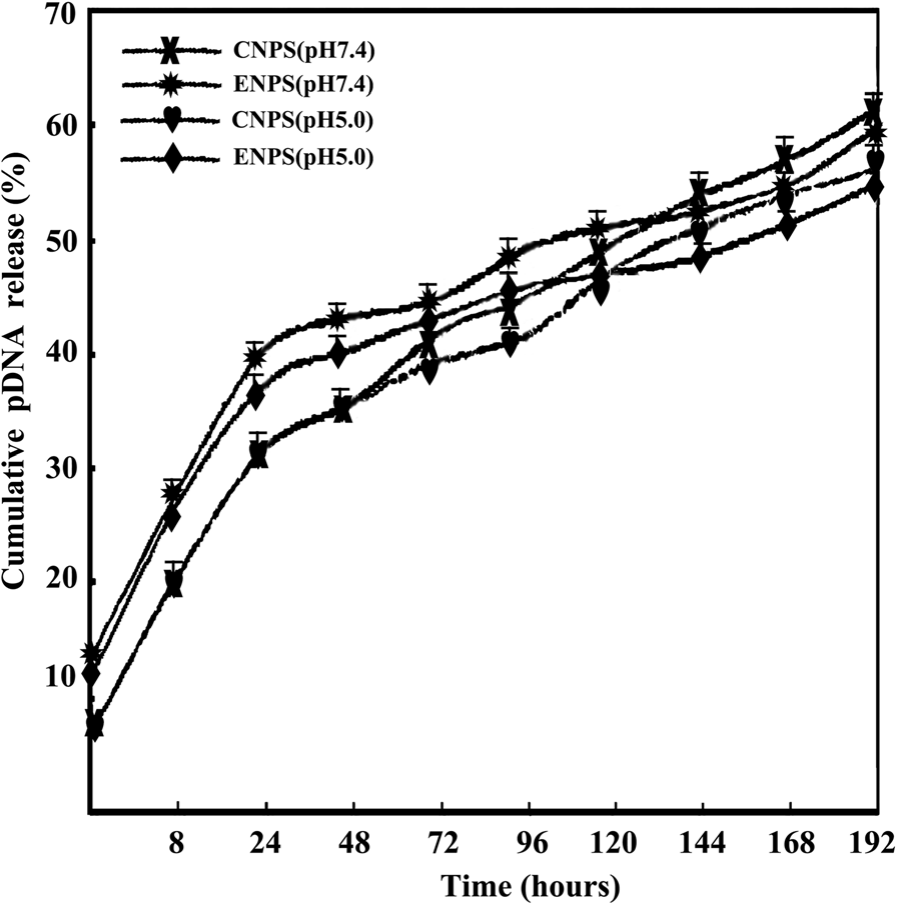
**The *in vitro* release profile of pDNA from various NPs at pH 7.4 and pH 5.0.**

Previous study showed that the kinetic of NP degradation and DNA release appears to have a significant regulatory effect on the gene expression [26]. In this study, we compared the *in vitro* release of pDNA from docetaxel-loaded or doceraxel-free TPGS-*b*-(PCL-*ran*-PGA)/PEI-pDNA (DNPs or ENPs) formulations at pH 7.4 and pH 5.0. To simulate acidification in the endo-lysosome of cells following intracellular uptake, NPs were first incubated in a solution at pH 7.4 for 24 h, then one-half of the NPs continued to be incubated at pH 7.4, and another half was transferred to a solution of pH 5.0. pDNA release was monitored by measuring UV absorption at 260 nm at predetermined time points. In the pH 7.4 group, 40.5% and 43.1% of the total pDNA were released rapidly from docetaxel-loaded NPs/PEI and empty NPs/PEI, respectively, at 48 h, followed by a slow release until day 7 (Figure 3). These two formulations had no significant difference either at initial burst phase or slow release phase at pH 5.0, suggesting that the loaded docetaxel had no significant influence on pDNA release. Zolnik's group reported that cleavage of the ester linkage of the PLGA polymer backbone at low pH can accelerate the polymer erosion [27]. However, in this study, the release kinetics of pDNA at pH 7.4 was similar to that at pH 5.0, but the release of pDNA from NPs from day 7 at pH 5.0 was more rapid than that at pH 7.4. The reason was the degradation of surface erosion [27]. Being a hydrophilic molecule located at the surface of the TPGS-*b*-(PCL-*ran*-PGA) matrix, PEI may facilitate the degradation of the NPs by increasing hydration and, thereby, accelerating hydrolysis [28].

**Figure 4:**
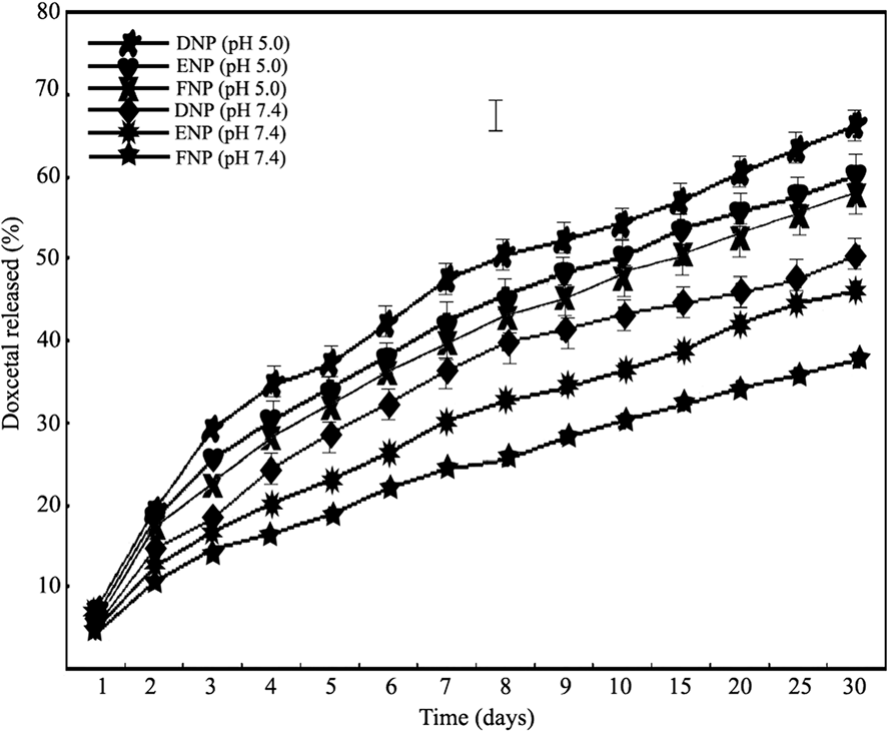
**The *in vitro* release curve of docetaxel from different NPs.**

In order to investigate the docetaxel release profiles, the three formulations of NPs, docetaxel-loaded TPGS-*b*-(PCL-*ran*-PGA), docetaxel-loaded TPGS-*b*-(PCL-*ran*-PGA)/PEI, and docetaxel-loaded TPGS-*b*-(PCL-*ran*-PGA)/ PEI-pDNA, were incubated in 37°C aqueous solutions at pH 7.4 and pH 5.0, respectively. As shown in Figure 4, a typical release profile was observed for all six tested nano-complex solutions, including an initial rapid release followed by a sustained slow release over a prolonged time up to several weeks. Furthermore, the docetaxel release at pH 5.0 was faster than the other nano-assemblies. Similar pH-dependent trend was also observed in other docetaxel-loaded polymeric micellar systems [29], and such behavior was assumed to improve intracellular drug release once the nano-complexes enter the tumor cells via endocytosis and trapped within the acidic endosomal/lysosomal compartments. In addition, both pDNA complexion and PEI coating were found to prolong docetaxel release, which is consistent with the results for a paclitaxel and Herceptin (Genentech, Inc., CA, USA) co-delivery system using cationic micellar NPs [30]. These results implied that the release profile of PEI-modified docetaxel-loaded TPGS-*b*-(PCL-*ran*-PGA) NPs was prolonged, and its toxicity might be decreased.

### Cellular uptake

**Figure 5:**
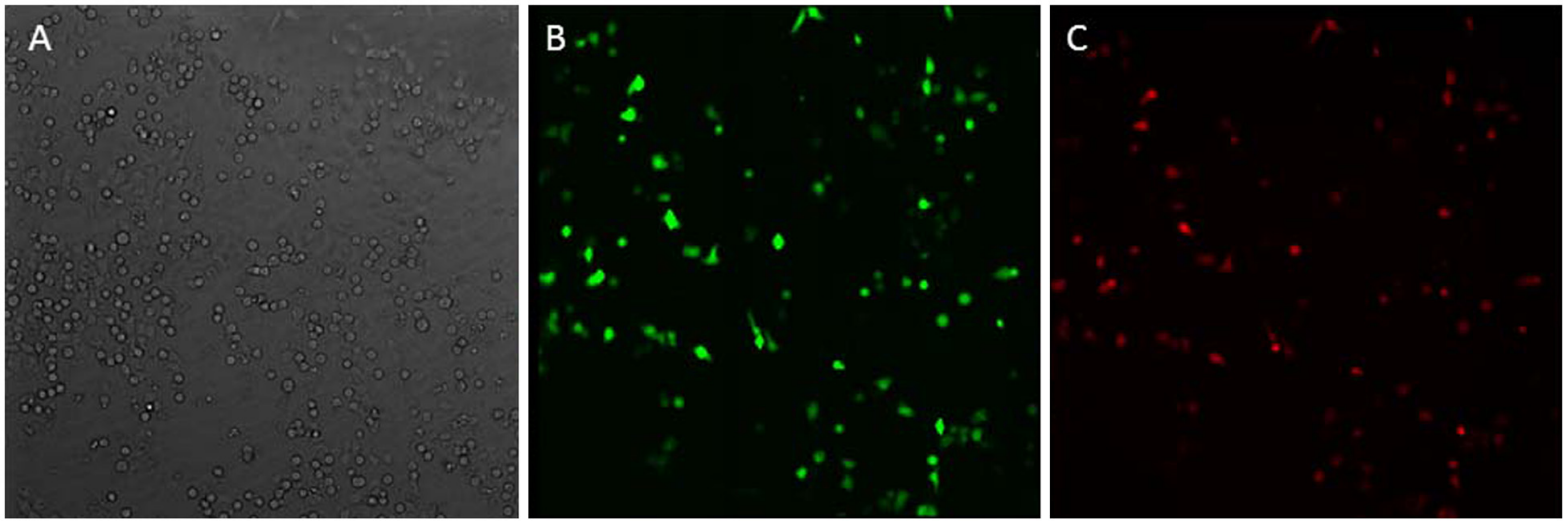
**The fluorescence microscopic images of 293T cells.** This is after the incubation with plasmids pIRES-EGFP-loaded and pdSRED-loaded TPGS-*b*-(PCL-*ran*-PGA)/PEI NPs; (**A**) from transmitted light channel, (**B**) from FITC channel, (**C**) from PI channel.

**Figure 6:**
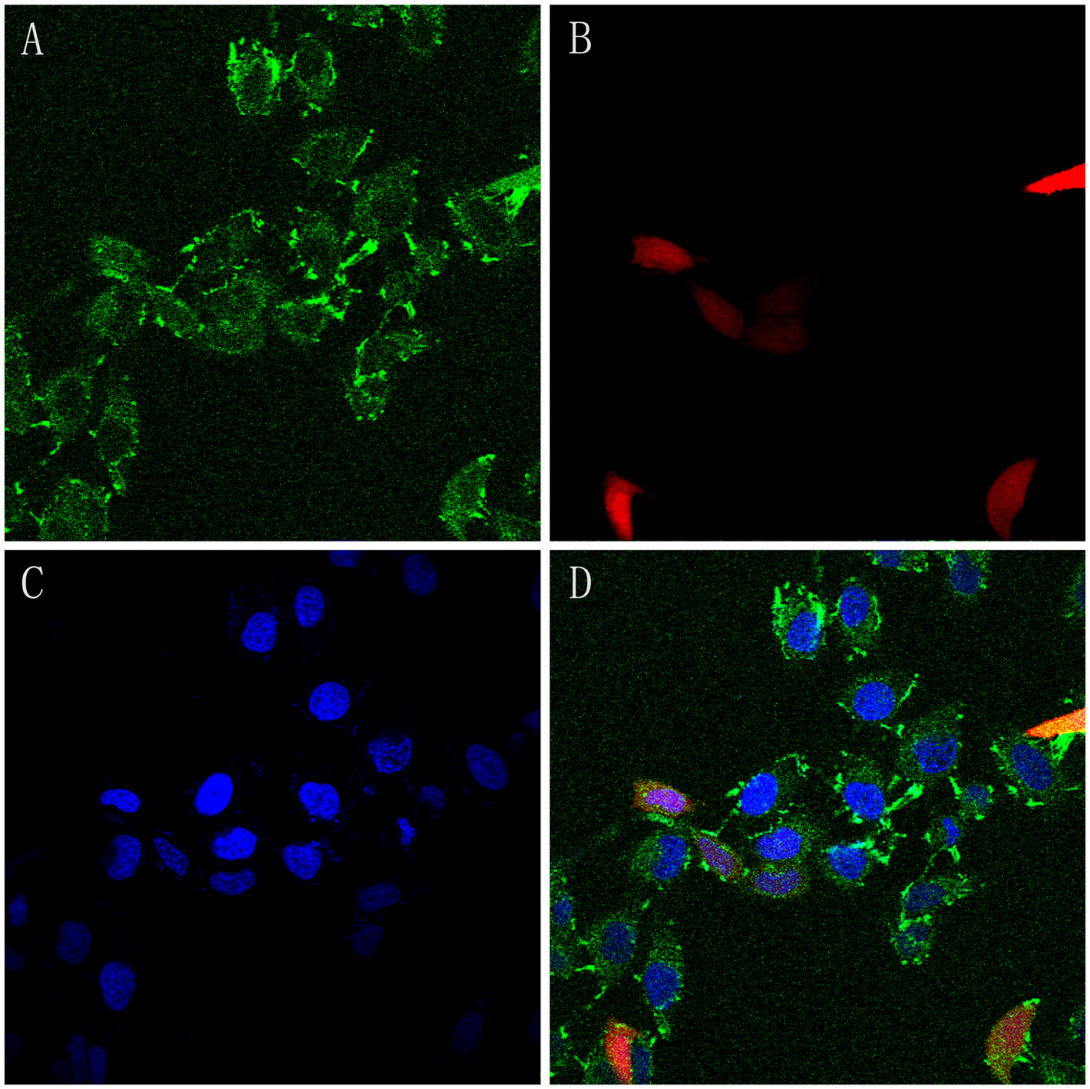
**Confocal laser scanning microscopy of HeLa cells.** This is after incubation with loaded-coumarin-6-TPGS-*b*-(PCL-*ran*-PGA)/PEI-pDSRED NPs at 37°C. The cells were stained by DAPI (blue), and the plasmids pDsRED-loaded-TPGS-*b*-(PCL-*ran*-PGA)/PEI NPs are red. The cellular uptake was visualized by overlaying images obtained. (**A**) Coumarin-6, (**B**) RED, (**C**) DAPI, and (**D**) co-merge.

To evaluate cellular uptake of nanoparticles, 293T or HeLa cells were incubated with PEI-modified TPGS-*b*-(PCL-*ran*-PGA) NPs and coumarin-6-loaded TPGS-*b*-(PCL-*ran*-PGA) NPs for 48 h. The fluorescence emitted by coumarin-6 and RED protein was observed after NP incubation, which suggested that the NPs efficiently entered the cells. The 293T cells took up the particles in a dose-dependent manner, and EGFP and RED expression achieved peak levels with 1.5 mg/ml of PEI complexed with 100 mg of NPs (Figure 5). These findings demonstrated that PEI-modified NPs may be good candidates for the delivering of genes into tumor cells, and PEI-modified TPGS-*b*-(PCL-*ran*-PGA) nanospheres may increase the gene binding ability which causes the endosomal escape easily and lowers the PEI cytotoxicity [25]. We also found that more PEI-modified coumarin-6-loaded NPs were uptaken by HeLa cells than unmodified NPs, which is in consistency with other reports [31]. It was probably because the PEI-modified NPs had positive charge on surface, which provided good affiliation with cells which had negative charge on the surface. In addition, our data also showed that coumarin-6 and RED protein can co-express in HeLa cells transfected by coumarin-6-loaded TPGS-*b*-(PCL-*ran*-PGA)/PEI-pDSRED (Figure 6), suggesting that PEI-modified TPGS-*b*-(PCL-*ran*-PGA) can co-deliver chemotherapeutic drugs and genetic materials into cells.

### Cell viability

**Figure 7:**
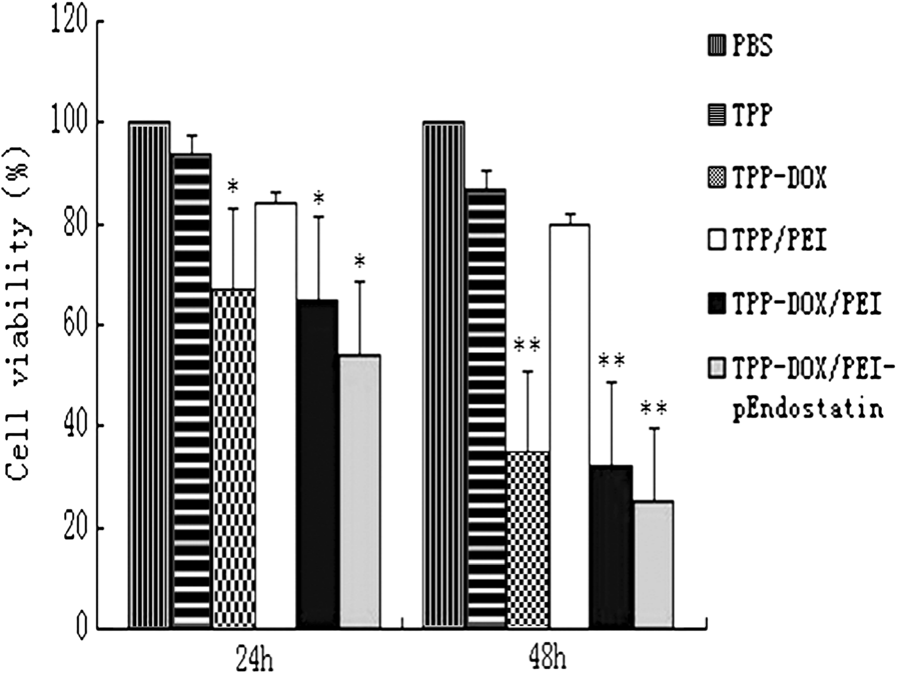
**Viability of HeLa cells after 24- and 48-h incubations (*n* = 5).** Single asterisk denotes *p* < 0.05 (compared with PBS), while double asterisk indicates *p* < 0.01 (compared with PBS).

Cytotoxicity of all NPs was determined in various concentrations using MTT assay. The cell viability was not affected by TPGS-*b*-(PCL-*ran*-PGA) NPs in all concentrations relative to the control (treated with PBS) after 72 h in medium. However, increasing dosage of PEI-modified TPGS-*b*-(PCL-*ran*-PGA) NPs was associated with increased cytotoxicity because of the increase of PEI (Figure 7). As expected, for TPGS-*b*-(PCL-*ran*-PGA), TPGS-*b*-(PCL-*ran*-PGA)/PEI NPs, and coumarin-6-loaded NPs, the cell survival rates were greater than 84%, whereas cell survival rate of the cells treated with equal dose of PEI was less than 70% (data not shown), suggesting that TPGS-*b*-(PCL-*ran*-PGA) could reduce the cytotoxicity of PEI (Figure 7). On the other hand, docetaxel-loaded TPGS-*b*-(PCL-*ran*-PGA)/PEI-pEndostatin NPs appeared to have more cytotoxicity than that of the other control NPs including docetaxel-loaded NPs; this may be because endostatin could induce cell apoptosis [32]. Among all NPs, docetaxel-load NPs/PEI-endostatin had the highest cytotoxicity.

The cell viability of HeLa cells transfected with docetaxel-loaded NPs was decreased after 6 h, which was dose- and time-dependent. These results indicated that PEI-modified TPGS-*b*-(PCL-*ran*-PGA) NPs can simultaneously delivery docetaxel and genetic materials into cancer cells and kill them.

### *In vivo* studies

**Figure 8:**
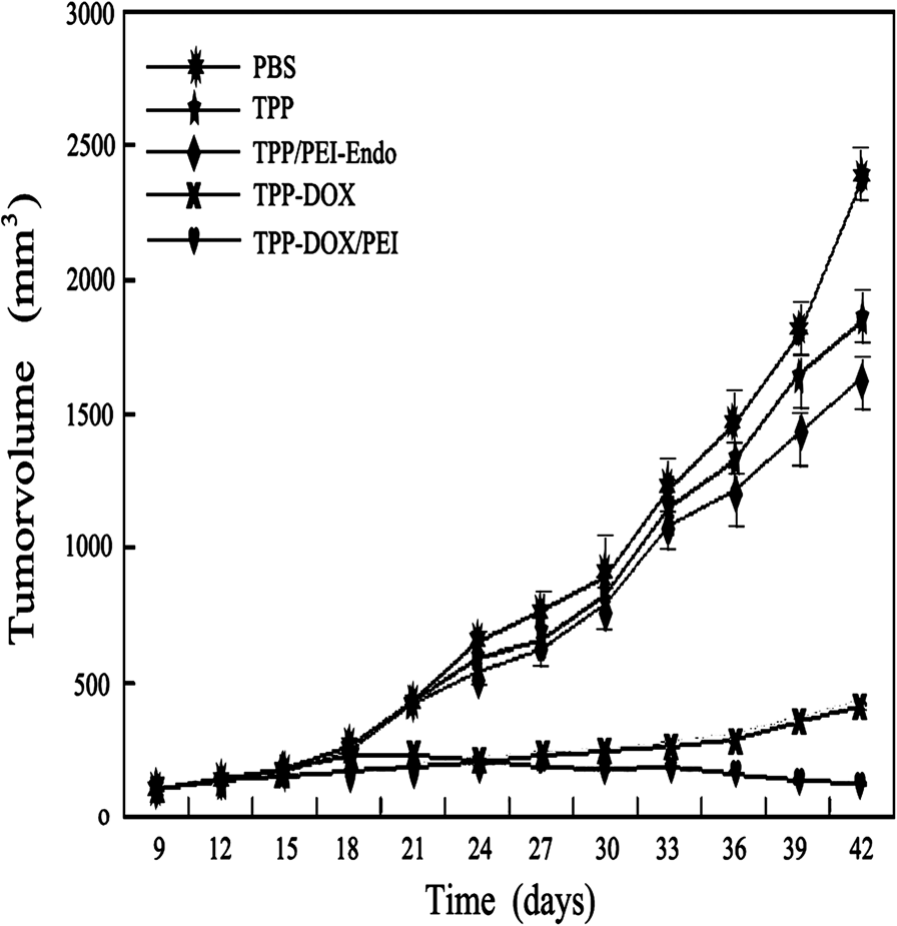
The tumor volume curve after animal models were treated by different NPs.

To investigate the therapeutic effect of TPGS-*b*-(PCL-*ran*-PGA)-based NPs on tumors, the anti-tumor efficacy and angiogenesis inhibition of various NPs including docetaxel-loaded NPs-PEI/pEndostatin were further evaluated *in vivo*. Subcutaneous tumor models were constructed in nude mice by being inoculated with 1 to 2 × 10^6^ HeLa cells. The results showed that CNPs, ENPs, and FNPs containing docetaxel significantly inhibited tumor growth compared with PBS, ANPs, and DNPs (*p* < 0.01) (Figure 8). The tumor volume of the mice treated with FNPs appeared to keep growing in the initial 5 days after treatment, and then the tumor sites presented inflammatory-like response and ulcer. After 3 weeks, the tumor growth was significantly inhibited, and 80% tumor was eradicated. Similar trend was also found in the groups treated with CNPs and ENPs, the inhibitory effect was better than other groups except of FNP treatment group. Treatment of mice with docetaxel-loaded NPs and PEI-modified docetaxel-loaded NPs inhibited 84% and 87% in tumor growth inhibition efficiency, respectively, despite no significant difference (*p* > 0.05).

**Figure 9:**
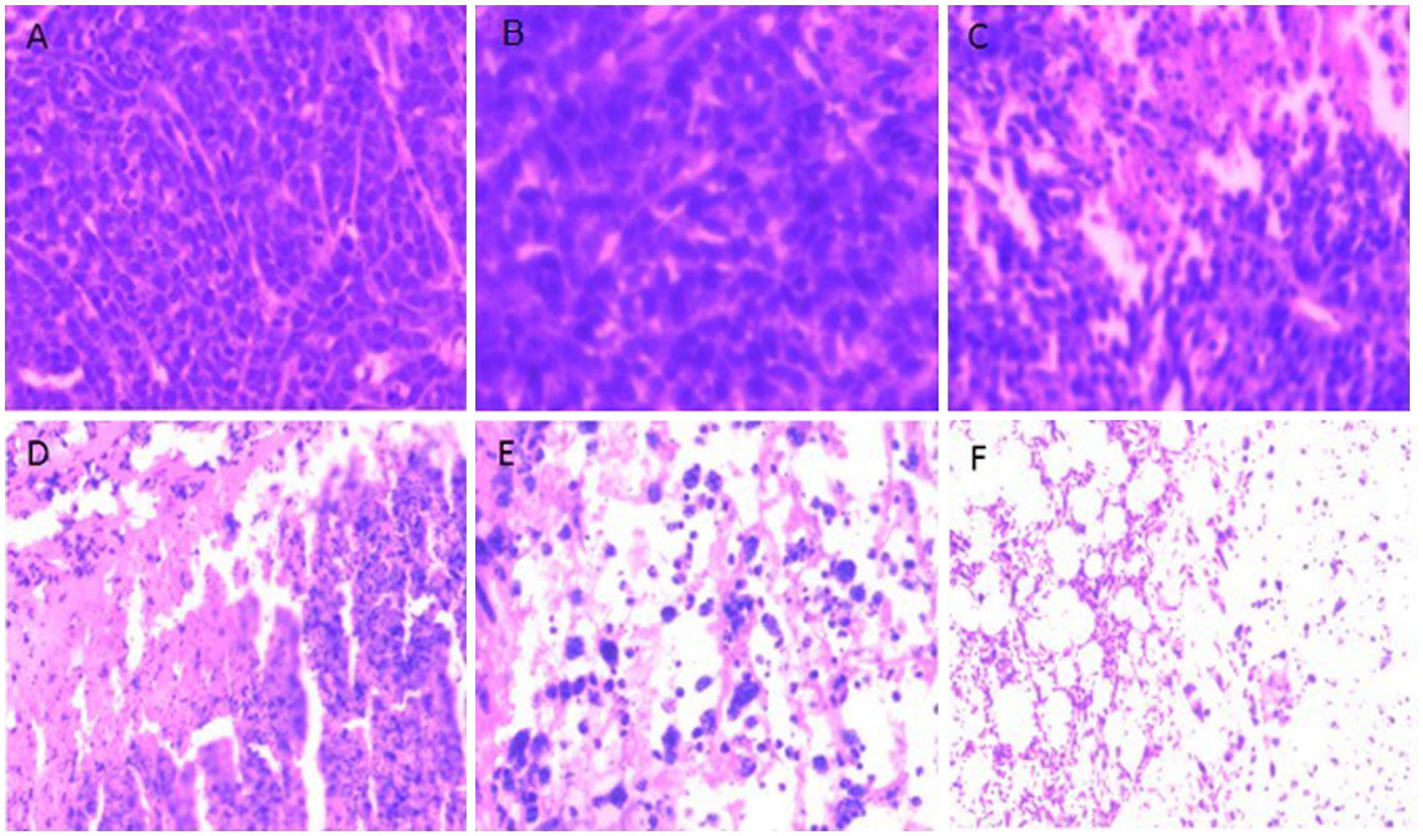
**The histopathological analysis of the tumor tissues from different treatment groups.** (**A**) PBS, (**B**) ANPs (TPGS-*b*-(PCL-*ran*-PGA)), (**C**) CNPs (TPGS-*b*-(PCL-*ran*-PGA/PEI/pEndo), (**D**) DNPs, (**E**) ENPs, and (**F**) FNPs.

**Figure 10:**
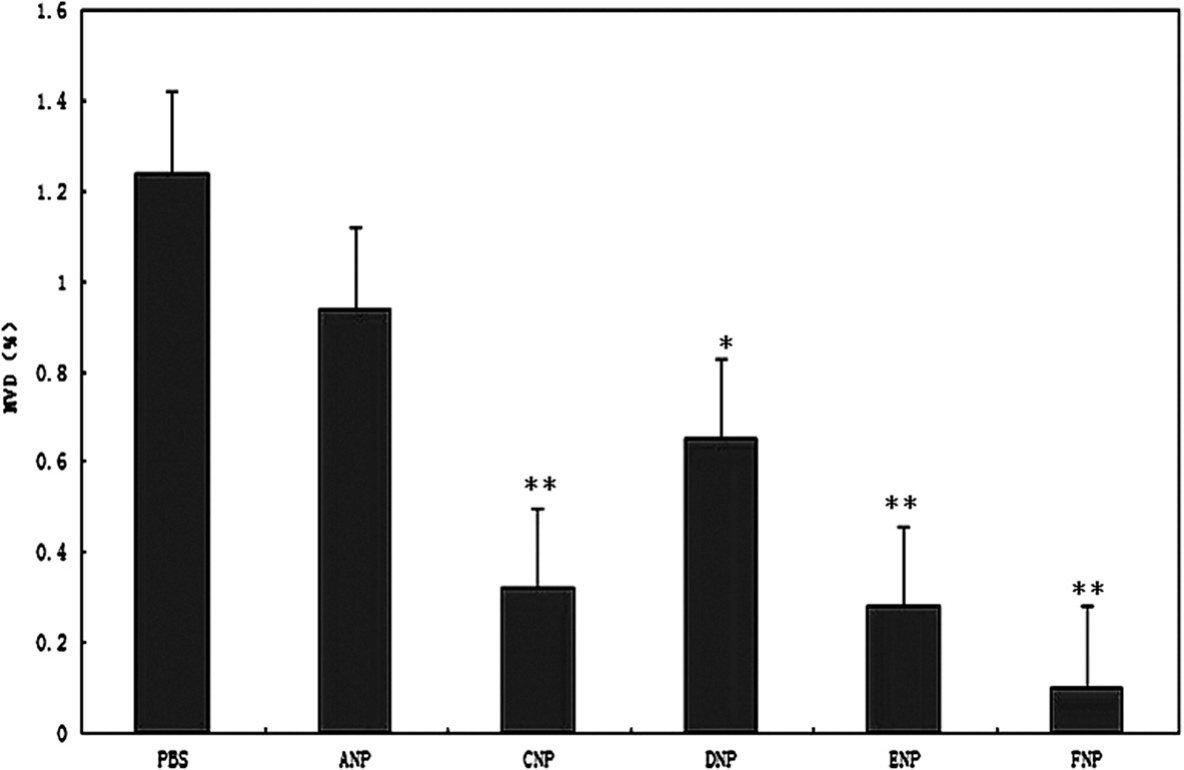
**MVD of tumor tissue in different treatment groups.** Single asterisk indicates *p* < 0.05 (compared with PBS), while double asterisk denotes *p* < 0.01 (compared with PBS).

Compared with the PBS group, the tumor growth inhibition of the groups treated with empty NPs and NPs/PEI-pEndostatin was 10% and 38%, respectively, which again proved that endostatin could inhibit tumor growth. The inhibitory effect of empty NPs may be caused by the TPGS degradation from the NP matrix (Figure 8). The histopathological analysis of the tumor tissues also revealed that NPs containing docetaxel or/and endostatin induced cell necrosis and apoptosis, leading to suppression of tumor progression (Figure 9). MVD in FNP, DNP, and CNP groups was relatively lower (*p* < 0.01) (Figure 10) compared with PBS group. MVD in DNP group was also lower in contrast with PBS group (*p* < 0.05) (Figure 10). This is due to the cell necrosis and apoptosis caused by docetaxel and endostatin. These results are consistent with histopathological analysis of the tumor tissues. No significant difference in mouse weight was observed among each NP treatment group (data not shown). At the end of the experiment, dissection results revealed that there were no obvious signs of toxicity in the heart, liver, lungs, spleen, kidneys, and other organs in each group. These results implied that TPGS-*b*-(PCL-*ran*-PGA)-based NPs could serve as multifunctional carrier of docetaxel and endostatin. In addition, during the regimen process, the release of TPGS may help the anticancer effect of docetaxel and endostatin because TPGS possesses the functions to improve drug permeability, enhance absorption of drugs, and reduce P-glycoprotein-mediated multi-drug resistance in cancer cells by inhibition of P-glycoprotein activity [33,34].

## Conclusions

In this research, PEI-modified docetaxel-loaded TPGS-*b*-(PCL-*ran*-PGA) NPs polyplexed with endostatin gene showed an optimizing potential in delivering genes and chemotherapeutic drugs. The co-delivery of endostatin and docetaxel by PEI-modified TPGS-*b*-(PCL-*ran*-PGA) NPs significantly inhibited the tumor growth of nude mouse models (even regress 80% established tumor). In conclusion, our study suggested that PEI-modified TPGS-*b*-(PCL-*ran*-PGA) NPs could function as an optimizing carrier for chemotherapeutic drugs and genetic materials.

## Competing interests

The authors declare that they have no competing interests.

## Authors’ contributions

BQ carried out the *in vivo* studies and drafted the manuscript. MJ carried out cell studies. XS carried out the preparation of nanoparticles. YoZ carried out characterization of nanoparticles. ZW carried out the *in vitro* drug release studies. XZ participated in the *in vivo* studies. SW participated in the cell studies. HC participated in the preparation of the nanoparticles. LM participated in the design of the study and performed the statistical analysis. YiZ conceived the study and participated in its design and coordination. All authors read and approved the final manuscript.

## Authors’ information

BQ is an associate chief physician of the Department of Orthopedics, Renmin Hospital of Wuhan University. MJ is a Ph.D. student of the Southern Medical University. XS is a Postdoctoral Fellow, Tsinghua University. YoZ, ZW, XZ, and SW are Ph.D. students of Tsinghua University. HC and YiZ are assistant professors of the Tsinghua University. LM is an associate professor of the Tsinghua University.

## References

[B1] IARC Monographs on the evaluation of carcinogenic risks to humans: human papillomaviruses, vol. 90 http://monographs.iarc.fr/

[B2] WHO (World Health Organization) Human papillomaviruses http://www.who. int/vaccine_research/ diseases/viral_cancers/en/index3.html

[B3] Schiller JT Castellsagué X Villa LL Hildesheim A An update of prophylactic human papillomavirus L1 virus-like particle vaccine clinical trial results Vaccine 2008 26 Suppl 10 K53 61 18847557 10.1016/j.vaccine.2008.06.002 PMC2631230

[B4] Winlaw DS Angiogenesis in the pathobiology and treatment of vascular and malignant diseases Ann Thorac Surg 1997 64 1204 1211 10.1016/S0003-4975(97)00716-9 9354565

[B5] Folkman J Is angiogenesis an organizing principle in biology and medicine? J Pediatr Surg 2007 42 1 11 10.1016/j.jpedsurg.2006.09.048 17208533

[B6] Folkman J Antiangiogenesis in cancer therapy-endostatin and its mechanisms of action Exp Cell Res 2006 312 594 607 10.1016/j.yexcr.2005.11.015 16376330

[B7] Folkman J Angiogenesis: an organizing principle for drug discovery? Nat Rev Drug Discov 2007 6 273 286 10.1038/nrd2115 17396134

[B8] Michael SO Antiangiogenesis and vascular endothelial growth factor/vascular endothelial growth factor receptor targeting as part of a combined-modality approach to the treatment of cancer Int J Radiat Oncol Biol Phys 2007 69 S64 S66 17848299 10.1016/j.ijrobp.2007.04.093 PMC2092454

[B9] O'Reilly MS Boehm T Shing Y Fukai N Vasios G Lane WS Flynn E Birkhead JR Olsen BR Folkman J Endostatin: an endogenous inhibitor of angiogenesis and tumor growth Cell 1997 88 277 285 10.1016/S0092-8674(00)81848-6 9008168

[B10] Boehm T Folkman J Browder T O'Reilly MS Antiangiogenic therapy of experimental cancer does not induce acquired drug resistance Nature 1997 390 404 407 10.1038/37126 9389480

[B11] Herbst RS Lee AT Tran HT Abbruzzese JL Clinical studies of angiogenesis inhibitors: the University of Texas MD Anderson Center Trial of Human Endostatin Curr Oncol Rep 2001 3 131 140 10.1007/s11912-001-0013-8 11177745

[B12] Zhuang HQ Yuan ZY Process in the mechanisms of endostatin combined with radiotherapy Cancer Letters 2009 282 9 13 10.1016/j.canlet.2008.12.008 19136200

[B13] Alexander BL Ali RR Alton EW Bainbridge JW Braun S Cheng SH Flotte TR Gaspar HB Grez M Griesenbach U Kaplitt MG Ott MG Seger R Simons M Thrasher AJ Thrasher AZ Ylä-Herttuala S Progress and prospects: gene therapy clinical trials (part 1) Gene Ther 2007 20 1439 1447 17909539 10.1038/sj.gt.3303001

[B14] Guinn BA Mulherkar R International progress in cancer gene therapy Cancer Gene Ther 2008 12 765 775 18846114 10.1038/cgt.2008.66

[B15] Scherer L Rossi JJ Weinberg MS Progress and prospects: RNA-based therapies for treatment of HIV infection Gene Ther 2007 14 1057 1064 10.1038/sj.gt.3302977 17607313

[B16] Androic I Krämer A Yan R Rödel F Gätje R Kaufmann M Strebhardt K Yuan J Targeting cyclin B1 inhibits proliferation and sensitizes breast cancer cells to taxol BMC Cancer 2008 8 391 401 10.1186/1471-2407-8-391 19113992 PMC2639606

[B17] Gao Y Liu XL Research progress on siRNA delivery with nonviral carriers Int J Nanomedicine 2011 6 1017 1025 21720513 10.2147/IJN.S17040 PMC3124387

[B18] Zhou J Wu J Hafdi N PAMAM dendrimers for efficient siRNA delivery and potent gene silencing Chem Commun (Camb) 2006 22 2362 2364 16733580 10.1039/b601381c

[B19] Mao SR Sun W Kissel T Chitosan-based formulations for delivery of DNA and siRNA Adv Drug Deliv Rev 2010 62 12 27 10.1016/j.addr.2009.08.004 19796660

[B20] Son S Kim WJ Biodegradable nanoparticles modified by branched polyethylenimine for plasmid DNA delivery Biomaterials 2010 31 133 143 10.1016/j.biomaterials.2009.09.024 19783041

[B21] Blum JS Saltzman WM High loading efficiency and tunable release of plasmid DNA encapsulated in submicron particles fabricated from PLGA conjugated with poly-l-lysine J Control Release 2008 129 66 72 10.1016/j.jconrel.2008.04.002 18511145 PMC2494593

[B22] Boussif O Lezoualc'h F Zanta MA Mergny MD Scherman D Demeneix B Behr JP A versatile vector for gene and oligonucleotide transfer into cells in culture and in vivo: polyethylenimine Proc Natl Acad Sci USA 1995 92 7297 7301 10.1073/pnas.92.16.7297 7638184 PMC41326

[B23] Zhu LP Xing J Wang QX Kou L Li C Hu B Wu ZW Wang JJ Xu GX Therapeutic efficacy of recombinant human endostatin combined with chemotherapeutics in mice-transplanted tumors Eur J Pharmacol 2009 617 23 27 10.1016/j.ejphar.2009.07.003 19615993

[B24] Huang L Chen H Zheng Y Song X Liu R Liu K Zeng X Mei L Nanoformulation of d-α-tocopheryl polyethylene glycol 1000 succinate-b-poly(ε-caprolactone-ran-glycolide) diblock copolymer for breast cancer therapy Integr Biol 2011 3 993 1002 10.1039/c1ib00026h 21938302

[B25] Kim JH Park JS Yang HN Woo DG Jeon SY Do HJ Lim HY Kim JM Park KH The use of biodegradable PLGA nanoparticles to mediate SOX9 gene delivery in human mesenchymal stem cells (hMSCs) and induce chondrogenesis Biomaterials 2011 32 268 278 10.1016/j.biomaterials.2010.08.086 20875683

[B26] Wang C Ge Q Ting D Nguyen D Shen HR Chen J Eisen HN Heller J Langer R Putnam D Molecularly engineered poly (ortho ester) microspheres for enhanced delivery of DNA vaccines Nat Mater 2004 3 190 196 10.1038/nmat1075 14991022

[B27] Zolnik BS Burgess DJ Effect of acidic pH on PLGA microsphere degradation and release J Control Release 2007 122 338 344 10.1016/j.jconrel.2007.05.034 17644208

[B28] Yin Y Chen D Qiao M Wei X Hu H Lectin-conjugated PLGA nanoparticles loaded with thymopentin: ex vivo bioadhesion and in vivo biodistribution J Control Release 2007 123 27 38 10.1016/j.jconrel.2007.06.024 17728000

[B29] Kataoka K Matsumoto T Yokoyama M Okano T Sakurai Y Fukushima S Okamoto K Kwon GS Doxorubicin-loaded poly(ethylene glycol)-poly(beta-benzyl-L-aspartate) copolymer micelles: their pharmaceutical characteristics and biological significance J Control Release 2000 64 143 153 10.1016/S0168-3659(99)00133-9 10640653

[B30] Lee AL Wang Y Cheng HY Pervaiz S Yang YY The co-delivery of paclitaxel and Herceptin using cationic micellar nanoparticles Biomaterials 2009 30 919 927 10.1016/j.biomaterials.2008.10.062 19042015

[B31] Fay F Quinn DJ Gilmore BF McCarron PA Scott CJ Gene delivery using dimethyldidodecylammonium bromide-coated PLGA nanoparticles Biomaterials 2011 31 4214 4222 20185174 10.1016/j.biomaterials.2010.01.143

[B32] Wang L Yao B Li Q Mei K Xu JR Li HX Wang YS Wen YJ Wang XD Yang HS Li YH Luo F Wu Y Liu YY Yang L Gene therapy with recombinant adenovirus encoding endostatin encapsulated in cationic liposome in coxsackievirus and adenovirus receptor-deficient colon carcinoma murine models Hum Gene Ther 2011 22 9 1061 1069 10.1089/hum.2011.014 21615297

[B33] Collnot EM Baldes C Wempe MF Kappl R Hüttermann J Hyatt JA Edgar KJ Schaefer UF Lehr CM Mechanism of inhibition of P-glycoprotein mediated efflux by vitamin E TPGS: influence on ATPase activity and membrane fluidity Mol Pharm 2007 4 3 465 474 10.1021/mp060121r 17367162

[B34] Dintaman JM Silverman JA Inhibition of P-glycoprotein by Dalpha-tocopheryl polyethylene glycol 1000 succinate (TPGS) Pharm Res 1999 16 1550 1556 10.1023/A:1015000503629 10554096

